# Foraging responses of bumble bees to rewardless floral patches: importance of within-plant variance in nectar presentation

**DOI:** 10.1093/aobpla/plw037

**Published:** 2016-07-11

**Authors:** Shoko Nakamura, Gaku Kudo

**Affiliations:** ^1^Graduate School of Environmental Science, Hokkaido University, Kita-ku N10 W5, Sapporo, Hokkaido, 060-0810, Japan; ^2^Faculty of Environmental Earth Science, Hokkaido University, Kita-ku N10 W5, Sapporo, Hokkaido, 060-0810, Japan

**Keywords:** Aconites, bumble bees, geitonogamy, plant distribution, pollination, rewardless, variation in nectar secretion

## Abstract

Spatiotemporal variation in nectar distribution is a key factor affecting pollinator movements between flowers and plants within a population. Pollinators having systematic searching ability can flexibly respond to the reward condition of floral patches, and they tend to revisit rewarding patches. However, foraging behaviour may be influenced by the nectar distribution within populations. To evaluate the effects of unrewarding experiences and plant distribution, we compared bumble bee foraging behaviours between naturally rewarding and artificially rewardless (by nectary removal) patches in two aconite populations with different plant densities. Visitation frequency to the patches, number of successive flower visits within inflorescences, and successive inflorescence visits within patches were recorded. Nectar production and standing crop were also measured. Bumble bees increased the movements between neighbouring inflorescences instead of leaving the patches when they faced rewardless flowers. A large variance in nectar production existed among flowers within plants. This might explain the observed bumble bee behaviour, because they could be rewarded by moving to the adjacent inflorescences even after a rewardless experience. Our results imply that a highly variable nectar reward in a population might mask the disadvantage of completely rewardless individuals.

## Abstract (precis)

Nectar foraging pollinators flexibly respond to the reward condition of floral patches. To evaluate the effects of unrewarding experience, we compared foraging behaviours of bumble bees between naturally rewarding and artificially rewardless (i.e., nectary removed) patches in aconite populations. Bees increased movements between inflorescences instead of leaving the patches when they faced rewardless flowers. Because the nectar reward was highly variable among flowers within plants in the aconite populations, they could be rewarded by the adjacent inflorescences even after unrewarding experiences. Completely rewardless plants might be pollinated successfully in rewarding populations if surrounding plants provide a highly variable nectar reward.

## Introduction

The effect of nectar presentation by plants on pollinator behaviour is a key aspect for pollination success (Real 1983; Chittka and Thomson 2001; Willmer 2011). Bumble bees, major pollinators in the cool-temperate ecosystems, use memory of past experiences to efficiently collect nectar. They tend to visit high quality patches repeatedly (Chittka and Thomson 2001; Goulson 2010). However, reward in flowers often varies unpredictably, reflecting biotic and abiotic conditions as well as inherent traits of plants (Antoń and Denisow 2014; reviewed in Goulson 1999; Mitchell 2004). As a result, pollinators flexibly respond to the heterogeneous resource conditions. They tend to leave an inflorescence or a patch quickly when reward is highly variable (risk aversion; Real 1981; Biernaskie *et al.* 2002; Hirabayashi *et al.* 2006). Area-restricted searching is also reported; that is, pollinators fly shorter distances between flowers when they encounter high-reward flowers but longer distances after an experience of low-reward flowers (Heinrich 1979; Baum and Grant 2001; Burns and Thomson 2005).

Inclusion of empty flowers within a rewarding floral patch may be advantageous for plants if risk-averse behaviour of pollinators can reduce geitonogamy and pollen discounting (Smithson and Gigord 2003; Biernaskie and Cartar 2004; Ishii *et al.* 2008). In addition, empty flowers can save the energetic cost of nectar production (Ordano and Ornelas 2005). About 7500 animal pollinated species are reported to be completely rewardless (reviewed in Renner 2006). Even rewardless species can benefit from the surrounding rewarding ones for pollinator attraction when rewardless plants are rare compared with rewarding ones (Johnson *et al.* 2003; Juillet *et al.* 2007).

Population structure also affects foraging behaviour of pollinators. Plant density and spatial distribution of patches are important factors determining pollinator movements within a population (Cresswell 2000; Ohashi and Yahara 2002; Grindeland *et al.* 2005; Geslin *et al.* 2014). For example, frequent movements between patches and a small number of successive flower visits in each patch are expected in a dense population (i.e. marginal value theorem; Charnov 1976). Furthermore, plant population structure may affect behaviours of pollinators. Although pollinators having trapline foraging behaviour regularly visit a set of floral patches (Ohashi *et al.* 2007), the frequency of occasional visits to focal patches tends to increase in a dense population (Makino *et al.* 2007).

From these findings, we expected that the responses of pollinators to completely rewardless plants might differ between populations with different plant distribution. However, population-scale studies on pollination success of rewardless plants are limited, in cases where rewardless individuals coexist with rewarding conspecifics (but see López-Portillo *et al.* 1993). In this study, we evaluated the effects of unrewarding experiences on bumble bee foraging behaviours in aconite populations. We artificially established rewardless patches in two populations having different plant density and habitat type; one was a dense population located in a grassland and the other was a sparse population under a closed forest. Specifically, we predicted the following: (i) total flower visits within patches will be smaller at the rewardless patches compared with the naturally rewarding ones; (ii) departure threshold in the successive rewardless-flower visits within patches will be smaller in the dense grassland population because of lower traveling cost; (iii) visitation frequency will decline in the rewardless patches if bumble bees can learn the location, but this trend may be less clear in the dense grassland population if the ratio of occasional visits is higher in the grassland. We also examined the source of variation in nectar production in both populations. We examined these predictions, and discussed the resulting pollination success of rewardless plants in each habitat.

## Methods

### Materials

*Aconitum sachalinense* subsp. *yezoense* (hereafter aconite) is a perennial herb distributed in Hokkaido, Japan. They grow in meadows and forests in which populations are composed of scattered patches. Flowering lasts for 2 months (primarily August and September), during which individual plants produce several inflorescences with racemed flower arrangements. Most of the inflorescences are displayed horizontally because of the curvature in the main stem structure. This architecture results in a spatially mixed arrangement of inflorescences from different plants. The zygomorphic flowers have two petals covered by a posterior helmet-shaped calyx, and the nectaries are located at the curved end of the tubular petals. Flowers are protandrous hermaphrodite without an overlap between long male and short female phases. Long-tongued bumble bees, *Bombus diversus tersatus* and *Bombus* *yezoensis* are the major foragers for nectar.

### Study sites

We selected two sites of aconite populations in 2010 at Jozankei in Hokkaido, that were separated by ∼1.9 km; one was on the floor of a deciduous forest (42°59′31″N, 141°06′36″E, 354 m elevation) and the other was in a grassland (42°58′56″N, 141°07′44″E, 328 m elevation). There was no other aconite population between the sites. In the forest site, nine small patches composed of a few plants were sparsely scattered within a 23 × 16 m^2^ area. In the grassland site, there were two medium-sized patches and a large dense patch, which was composed of >150 plants within a 22 × 12 m^2^ area. Flowering of aconite started on 25 July and 14 August in the grassland and forest sites, respectively, and it finished by 5 October in both sites. The peak flowering period was from 14 to 30 August and 25 August to 12 September in the grassland and forest sites, respectively. The inflorescence density per 1 m^2^ was higher in the grassland site (12.1 ± 4.1 (mean ± SD), *n* = 20 in the forest and 20.9 ± 8.2, *n* = 17 in the grassland; *z* = 6.62, *P* < 0.001 by generalized linear mixed effect model (GLMM)), whereas the number of open flowers within inflorescences did not differ (4.5 ± 1.2, *n* = 13 in the forest and 4.4 ± 1.0, *n* = 13 in the grassland; *z* = −0.09, *P* = 0.93). We observed 366 visits of *B. diversus tersatus* (349 workers, 11 males and 6 queens), 17 visits of *B. yezoensis* (5 workers, 4 males and 8 queens), and 5 visits of *Bombus* *hypocrita sapporoensis* (nectar robbing workers) on aconite flowers during 1390 min over 9 days at the peak flowering. Average foraging densities at aconite flowers (i.e. the number of bumble bee visits to 25 m^2^ per 90 min) were 43.0 ± 35.9 (*n* = 3) and 16.7 ± 9.6 (*n* = 3) in the grassland and forest sites, respectively. Aconite was most abundant and preferred by *B. diversus tersatus* among three and six co-flowering species at the forest and grassland sites, respectively. The second preferred species were *Impatiens noli-tangere* and *Trifolium pratense* at the forest and grassland sites, respectively (unpubl. obs.).

### Bumble bee observations

We established two small patches of about 1 m^2^ in each site, the ‘rewardless patch’ in which nectaries of all flowers were removed and the ‘control patch’ in which all nectaries were left intact. The patches were randomly selected in the forest site, and established by partitioning a few individual plants from the large patch by artificially creating a 0.5-m inflorescence-less buffer zone in the grassland site. In the rewardless patches, we pulled up the petals from the helmet-shaped calyx, removed only the nectary using small scissors, and replaced the petals in the calyx without causing physical damage to the floral structure. Thus, foraging bumble bees were unable to visually detect the absence of nectaries unless they tested the flowers. Nectary removal was done in the morning of the observation days. We ensured that there were 50–60 flowers in each patch to determine the differences in foraging behaviour between the forest and the grassland sites (see Table 1 for patch structure).

**Table 1.** The numbers of open flowers, inflorescences and plants in each patch

Site	Treatment	25 August 2010			30 August 2010		
		Flower	Inflorescence	Plant	Flower	Inflorescence	Plant
Forest	Control	53	14	5	58	16	5
	Rewardless	52	12	1	52	8	1
Grassland	Control	51	12	3	51	12	7
	Rewardless	52	16	4	52	9	1

Foraging behaviours of bumble bees were observed for 2 days with good weather on 25 and 30 August 2010, during the peak flowering period in both sites. We counted the number of visited inflorescences and flowers per inflorescence for every bout of bumble bees. We did not identify bumble bee individuals, and bumble bees that arrived at the patches were considered independent. In supplemental observations in 2011; however, we measured visitation frequency at an individual base by marking **[see Supporting Information Fig. S1]**. The period of a single observation session was 90 min, and we repeated the sessions twice a day. The first session began 10–60 min following the nectary removal, and the second session began 90 min following the end of the first session. Sessions for control and rewardless patches were started at the same time in both sites by either direct observation or video recording. From these data, we obtained visitation frequency to patches during 90 min, the number of successive flower visits within inflorescences, and the numbers of successive inflorescence visits and total flower visits within patches during a single bout. To check the effect of nectar manipulation on foraging behaviour in a flower, we counted the number of labium extension as they probed nectaries through petals **[see Supporting Information Fig. S2]**.

### Flower and population characteristics

To determine the nectar standing crop during the observation in 2010, nectar volume was measured for 40 intact flowers selected randomly in each site. Nectar standing crop was determined for 10 flowers immediately after the 90-min session, and the measurement was repeated twice a day for 2 days. To assess nectar production, we randomly bagged 10 flowers in each site in the morning of the two observation days, and measured nectar volume 5 h following the bagging treatment. Nectar was extracted using 1 or 2 µL-Microcaps (Drummond Scientific Company, USA). Flowers for nectar measurement were selected outside of the observation patches. According to our preliminary measurement, nectar sugar concentration was higher in the grassland site (Wilcoxon rank sum test, *W* = 332.5, *P* < 0.01) **[see Supporting Information Fig. S3]**. Additional data on diel variation in nectar standing crop is available **[see Supporting Information Fig. S4]**.

To examine the variance of nectar production between populations, among plants within populations, within plants and between floral sex phases, we measured nectar volume per flower after one-night bagging treatment. For this purpose, 56 flowers (43 male- and 13 female-phase flowers) on 14 plants and 87 flowers (77 male- and 10 female-phase flowers) on 10 plants were randomly selected in the forest and grassland sites, respectively, during the peak flowering season in 2015.

### Statistical analysis

Visitation frequency was analyzed by generalized linear model (GLM) assuming a Poisson error distribution in which site, nectar treatment, and inflorescence number within patches (inflorescence density) were included as fixed factors. For the analyses of successive flower visits within inflorescences, successive inflorescence visits within patches, and total flower visits within patches, we assumed a negative binomial error distribution. In the model of successive flower visits, site, nectar treatment, and flower number per inflorescence were included as fixed factors. In the models of successive inflorescence visits and total flower visits, site and nectar treatment were included as fixed factors. Inflorescence density was also included as a fixed factor for the analysis of successive inflorescence visits.

To examine the differences in nectar production between floral sex phases (bagged flowers in 2015), we used GLMM assuming a gamma error distribution with log-link function in which site and sex phase were treated as fixed factors, and plant ID was included as a random factor. Variance components of nectar production were estimated by a two-level unbalanced nested analysis of variance (ANOVA) in which site was treated as a fixed factor and plant ID as a random factor. To evaluate the effects of these explanatory valuables and select the best-fit models, we used Akaike’s information criterion (AIC). Statistical analyses are performed using R version 3.1.2 (R Core Team 2014). Estimation of the variance components was based on McDonald (2014).

## Results

### Bumble bee observations

**Figure 1.**
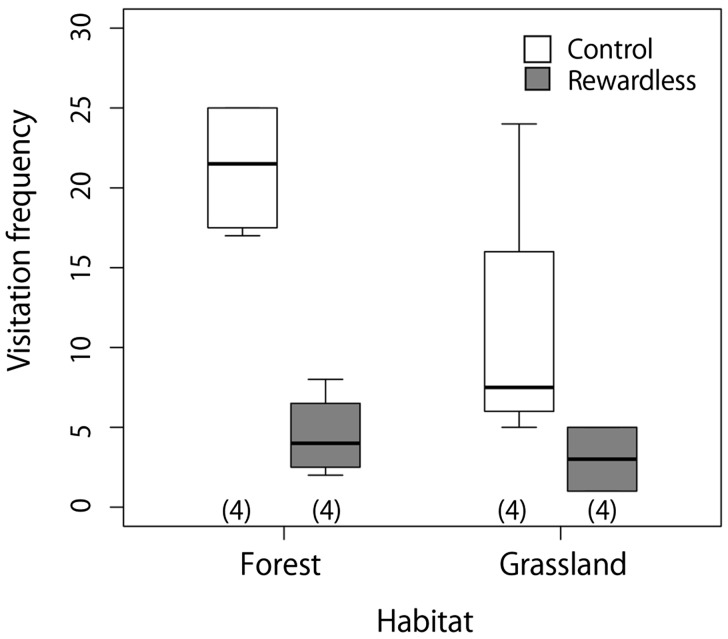
Visitation frequency of bumble bees to the control and rewardless patches. Grey boxes indicate rewardless patches and white indicate control patches. Figures in the parentheses represent sample sizes. The lower ends of the boxes represent the first quartiles and the upper the third quartiles, the segments inside the boxes indicate medians, and whiskers above and below the boxes indicate the minimums and maximums.

All visitors were *B. diversus tersatus* during the sessions in both sites. We observed 159 visits in total; most of the visits were by workers, and only 6 of them were by queens. Visitation frequencies to patches per 90 min were 21.3 ± 4.3 (mean ± SD) and 4.5 ± 2.6 at the control and rewardless patches in the forest site, respectively, and 11.0 ± 8.8 and 3.0 ± 2.3 in the grassland site, respectively (Fig. 1). Visitation frequency was significantly lower in the grassland site (*P* = 0.011), lower at the rewardless patches (*P* < 0.01), and tended to increase with inflorescence density (*P* = 0.087; Table 2). The interaction between site and treatment was excluded by AIC.

**Table 2.** Results of GLMs for visitation frequency, successive flower visits within inflorescences, successive inflorescence and total flower visits within patches. Model selection was carried out to maximize the goodness of fit given by AIC.

Response variable	Explanatory variable	*n*	AIC	Estimate	SE	*z* value	*Pr* (>|*z*|)
Visitation frequency to patches		16	96.6				
	Intercept (site = forest, treatment = control)			1.946	0.648	3.00	0.003
	Habitat (grassland)			−0.469	0.184	−2.54	0.011
	Treatment (rewardless)			−1.261	0.225	−5.60	<0.001
	Inflorescence density			0.075	0.043	1.71	0.087
Successive flower visits within inflorescences		675	2068.4				
	Intercept (site = forest, treatment = control)			0.346	0.082	4.21	<0.001
	Treatment (rewardless)			−0.172	0.070	−2.45	0.014
	Inflorescence size			0.090	0.018	4.99	<0.001
Successive inflorescence visits within patches		159	782.0				
	Intercept (site = forest, treatment = control)			0.446	0.459	0.97	0.331
	Treatment (rewardless)			0.320	0.166	1.93	0.054
	Inflorescence density			0.068	0.032	2.12	0.034
Total flower visits within patches		158	1011.1				
	Intercept (site = forest)			2.207	0.087	25.24	<0.001
	Habitat (grassland)			−0.146	0.148	−0.98	0.330

**Figure 2.**
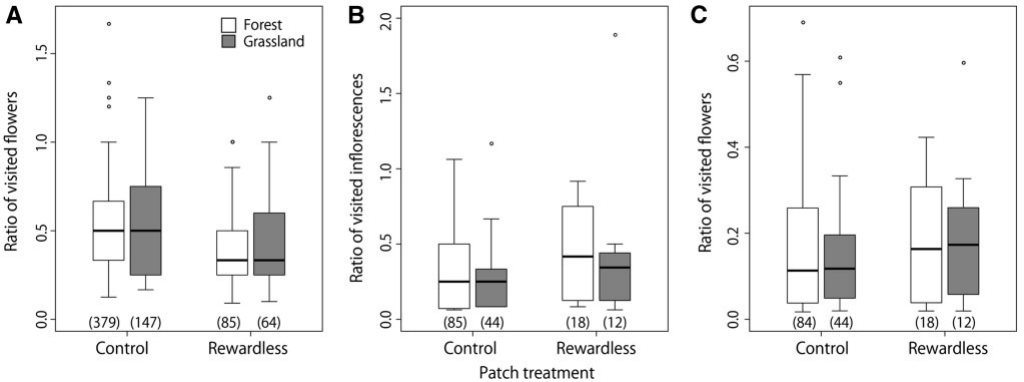
**(A)** The ratio of visited flowers to the total flowers per inflorescence (corresponds to successive flower visits within inflorescences), **(B)** The ratio of visited inflorescences to the total inflorescences per patch (corresponds to successive inflorescence visits within patches) and **(C)** The ratio of visited flowers to the total flowers per patch (corresponds to total flower visits within patches). Grey boxes indicate the grassland site, and white indicate the forest site. Figures in the parentheses represent sample sizes. The lower ends of the boxes represent the first quartiles and the upper the third quartiles, the segments inside the boxes indicate medians, whiskers above and below the boxes indicate the minimums and maximums within the range of 1.5 times of interquartile range from the upper or the lower quartiles, and outliers are represented by open circles.

Bumble bees visited 2.0 ± 1.1 flowers (54 % flowers per inflorescence) and 2.2 ± 1.3 flowers (51 %) within the control inflorescences in the forest and grassland sites, respectively. Within the rewardless inflorescences, bumble bees visited 2.0 ± 1.1 flowers (39 %) and 1.8 ± 1.2 flowers (43 %) in the forest and grassland sites, respectively (Fig. 2A). Bumble bees visited fewer flowers within inflorescences at the rewardless patches (*P* = 0.014) and more flowers in larger inflorescences (*P* < 0.01; Table 2), while site effect was excluded by AIC.

Bumble bees visited 4.5 ± 3.6 (30 % of total inflorescences) and 3.3 ± 2.9 (28 %) inflorescences within the control patches in the forest and grassland sites, respectively. Within the rewardless patches, bumble bees visited 4.7 ± 3.3 (44 %) and 5.3 ± 4.4 (42 %) inflorescences in the forest and grassland sites, respectively (Fig. 2B). Bumble bees visited more inflorescences at the rewardless patches (*P* = 0.054) and patches with higher inflorescence density (*P* = 0.034; Table 2), while site effect was excluded by AIC.

Bumble bees visited 9.0 ± 8.4 (16 % of total flowers) and 7.3 ± 6.6 (14 %) flowers before leaving the control patches in the forest and grassland sites, respectively. Within the rewardless patches, bumble bees visited 9.4 ± 7.0 (18 %) and 9.8 ± 8.6 (19 %) flowers at the forest and grassland sites, respectively (Fig. 2C). Bumble bees visited a similar proportion of flowers in both sites (*P* = 0.33; Table 2), and the effect of nectar treatment was excluded by AIC.

### Variance of nectar production and standing crop

Average nectar production of bagged flowers on the days of bumble bee observations was 0.42 ± 0.29 (mean ± SD) µL and 0.72 ± 0.57 µL in the forest and grassland sites, respectively. Nectar standing crops of intact flowers were 0.07 ± 0.14 and 0.31 ± 0.87 µL, including 31 (78 %) and 9 (23 %) flowers having no collectable nectar in the forest and grassland sites, respectively.

Nectar production of female-phase flowers was approximately three times larger than that of male phase flowers (*t* = 2.39, *P* = 0.017; one-night bagging in 2015). Female-phase flowers accumulated 1.68 ± 1.31 and 1.85 ± 1.87 µL nectar in the forest and grassland sites, respectively, whereas male-phase flowers accumulated 0.48 ± 0.71 and 0.62 ± 0.86 µL nectar, respectively. Among male-phase flowers, even under the bagging, about 14 % (6 flowers) and 19 % (15 flowers) produced no collectable nectar in the forest and grassland sites, respectively.

Because the frequency of male-phase flowers was three to eight times larger than female-phase flowers in both sites (likely because of the shorter female flowering period), the variance components of nectar productivity were estimated only for male-phase flowers. Within-plant variance accounted for 72 % of the overall variance in nectar production and the rest was caused by among-plant variance, whereas the between-site variance was estimated to be 0 (Table 3).

**Table 3.** Variance components of nectar production in aconite flowers. Variance components were estimated using a two-level unbalance nested ANOVA. *n* = 120 (43 flowers from the forest site and 77 from the grassland site).

	Sum of squares	df	Mean square	*Fs*	*P*	Variance component (%)
Among habitats	0.502	1	0.502	0.348	0.56	0
Individuals within habitats	30.314	21	1.444	3.014	0.00013	28.2
Within individuals	46.457	97	0.479			71.8
Total	77.273	119				100

## Discussion

Against the prediction made from the area-restricted searching, bumble bees visited similar number of flowers in both rewardless and control patches. Total flower visits in the rewardless patches were maintained by the frequent movements between inflorescences. In aconite, a large part of the variance of nectar production existed among flowers within plants. It implies the possibility of large differences among neighbouring inflorescences. *B. diversus tersatus* highly depend on aconite flowers for nectar resource in this season (unpubl. obs.). If they were familiar with the nectar distribution in aconite populations, they would obtain reward efficiently by moving to the adjacent inflorescences after a rewardless experience. Area-restricted searching is effective when the reward level varies among patches but is rather stable within patches (Thomson *et al.* 1982). This requirement might not be fulfilled in the aconite populations.

Although density and spatial distribution of plants often affect pollinator movements between plants (Cresswell 2000; Ohashi and Yahara 2002; Geslin *et al.* 2014), total flower visits within rewardless patches were independent of plant density in our observation. When a forager can evaluate a decelerating gain as it exploits the present patch, the decision of patch departure will be made comparing the expected reward gain at the patch and the travelling cost between patches (marginal value theorem; Charnov 1976). However, the large variation in nectar production among aconite flowers would make the estimation of reward gain within patches difficult. Thus, foragers might not behave in a manner predicted from the marginal value theorem.

As expected, visitation frequency to patches was significantly lower at the rewardless patches in both sites. We assumed that the bumble bees could not remotely discriminate the nectary-removed flowers. However, some insects use volatile cues released from plants (Schiestl 2010; Kessler *et al.* 2013). If nectary removal influenced the olfactory cues of aconite flowers, our treatment might decrease the attractiveness of rewardless patches. Although we could not exclude the possibility of the potential avoidance to the nectary-removed flowers, bumble bees seemed to decrease re-visitation to the rewardless patches after experiencing rewardless flowers **[see Supporting Information Fig. S1]**. Therefore, we predict that the experience of encountering rewardless flowers influenced subsequent behaviour of bumble bees. Although bumble bees might be confused by the internal structural change, they could learn the reward situation and respond to it (Makino and Sakai 2007).

We expected that the decrease in visitation frequency at rewardless patches would be mitigated in the grassland if occasional visits are more common in dense populations (Makino *et al.* 2007). However, our analysis did not suggest the mitigation effect because there was no interaction effect between site and treatment on visitation frequency.

### Significance of nectar distribution on pollination success

The large variance in reward within plants and the patch structure composed of multiple individuals might contribute to the outcrossing pollination of aconite. We observed a shorter stay in each inflorescence followed by frequent movements between neighbouring ones when bumble bees encountered rewardless flowers. This behaviour would ensure pollen receipt for many flowers, reducing the risk of geitonogamy (Biernaskie and Cartar 2004; Hirabayashi *et al.* 2006; Ishii *et al.* 2008). Bumble bees probed nectary-removed flowers similarly to the way they probed intact ones **[see Supporting Information Fig. S2]**. Although the time of stay in a flower might affect pollen receipt and removal (Thostesen and Olesen 1996; Kudo 2003), pollination efficiency might be less affected by the presence/absence of reward.

Completely rewardless plants might benefit from the large variance of nectar in surrounding conspecifics. Under the condition of highly variable reward gain in each flower, discrimination between completely rewardless flowers from transient ones would be difficult (Renner 2006). In this case, bumble bees would not be able to learn the location of completely rewardless plants. In aconite, 14–19 % of bagged male-phase flowers had no collectable nectar, and the ratio increased to 42–60 % in case of intact (i.e. unmanipulated) flowers **[see Supporting Information Fig. S4]**. Thus, large variance of reward in a population might mask the disadvantage of co-existing rewardless plants. As a result, once ‘magnet’ effects (Johnson *et al.* 2003; Peter and Johnson 2008) of surrounding population made enough number of bumble bees visit rewardless plants, rewardless plants would receive pollination service as rewarding ones do.

## Conclusions

Our results imply that even completely rewardless plants might be pollinated successfully in the rewarding population if surrounding plants provide a highly variable nectar reward to the pollinators. Thus, the effectiveness of the nectar presentation strategy of individual plants strongly depends on the spatial distribution of nectar reward at a local scale. This hypothesis can be tested by the comparisons of pollinator behaviour on rewardless patches between populations having high and low variance in nectar presentation. Furthermore, frequency and distribution of rewardless plants within a population, population size, and population density should be considered to evaluate the evolutionary significance of rewardless flowers.

## Sources of Funding

This work was partly supported by KAKENHI grant number 15H02641.

## Contributions by the Authors

Both of the authors conceived and designed the work, acquired the data, and analyzed and interpreted the data. S.N. drafted the work and both of the authors revised and approved the manuscript.

## Conflicts of Interest Statement

None declared.

## Supplementary Material

Supplementary Data

## Supporting Information

The following additional information is available in the online version of this article—

**Table S1.** Raw data of visitation frequency of identified bumble bees.

**Figure S1.** Boxplots of visitation frequency of identified bumble bees at the control and rewardless patches.

**Figure S2.** The number of labium extension as bumble bees probed nectaries through petals within flowers at the control (A) and rewardless (B) patches in the forest site, and the control (C) and rewardless (D) patches in the grassland site.

**Figure S3.** Variations in the nectar sugar concentration of *Aconitum sachalinense* flowers in the forest (A) and grassland (B) sites.

**Figure S4.** Diel variation in the nectar standing crop of intact flowers of *Aconitum sachalinense* in the forest (A) and grassland (B) sites.

## References

[plw037-B1] Antoń S Denisow B. 2014 Nectar production and carbohydrate composition across floral sexual phases: contrasting patterns in two protandrous *Aconitum* species (Delphinieae, Ranunculaceae). Flora-Morphology, Distribution, Functional Ecology of Plants 209:464–470.

[plw037-B2] Baum KA Grant WE. 2001 Hummingbird foraging behavior in different patch types: simulation of alternative strategies. Ecological Modelling 137:201–209.

[plw037-B3] Biernaskie JM Cartar RV Hurly TA. 2002 Risk averse inflorescence departure in hummingbirds and bumble bees: could plants benefit from variable nectar volumes? Oikos 98:98–104.

[plw037-B4] Biernaskie JM Cartar RV. 2004 Variation in rate of nectar production depends on floral display size: a pollinator manipulation hypothesis. Functional Ecology 18:125–129.

[plw037-B5] Burns JG Thomson JD. 2005 A test of spatial memory and movement patterns of bumblebees at multiple spatial and temporal scales. Behavioral Ecology 17:48–55.

[plw037-B6] Charnov EL. 1976 Optimal foraging, the marginal value theorem. Theoretical Population Biology 9:129–136. 1273796 10.1016/0040-5809(76)90040-x

[plw037-B7] Chittka L Thomson JD, eds. 2001 Cognitive ecology of pollination: animal behavior and floral evolution. Cambridge, UK: Cambridge University Press.

[plw037-B8] Cresswell JE. 2000 A comparison of bumblebees’ movements in uniform and aggregated distributions of their forage plant. Ecological Entomology 25:19–25.

[plw037-B9] Geslin B Baude M Mallard F Dajoz I. 2014 Effect of local spatial plant distribution and conspecific density on bumble bee foraging behaviour. Ecological Entomology 39:334–342.

[plw037-B10] Goulson D. 1999 Foraging strategies of insects for gathering nectar and pollen, and implications for plant ecology and evolution. Perspectives in Plant Ecology, Evolution and Systematics 2:185–209.

[plw037-B11] Goulson D. 2010 Bumblebees: behaviour, ecology, and conservation. USA: Oxford University Press.

[plw037-B12] Grindeland JM Sletvold N Ims RA. 2005 Effects of floral display size and plant density on pollinator visitation rate in a natural population of *Digitalis purpurea.* Functional Ecology 19:383–390.

[plw037-B13] Heinrich B. 1979 Resource heterogeneity and patterns of movement in foraging bumblebees. Oecologia 40:235–245. 10.1007/BF00345321 28309608

[plw037-B14] Hirabayashi Y Ishii HS Kudo G. 2006 Significance of nectar distribution for bumblebee behaviour within inflorescences, with reference to inflorescence architecture and display size. Ecoscience 13:351–359.

[plw037-B15] Ishii HS Hirabayashi Y Kudo G. 2008 Combined effects of inflorescence architecture, display size, plant density and empty flowers on bumble bee behaviour: experimental study with artificial inflorescences. Oecologia 156:341–350. 18283497 10.1007/s00442-008-0991-4

[plw037-B16] Johnson SD Peter CI Nilsson LA Ågren J. 2003 Pollination success in a deceptive orchid is enhanced by co-occurring rewarding magnet plants. Ecology 84:2919–2927.

[plw037-B17] Juillet N Gonzalez MA Page PA Gigord LDB. 2007 Pollination of the European food-deceptive *Traunsteinera globosa* (Orchidaceae): the importance of nectar-producing neighbouring plants. Plant Systematics and Evolution 265:123–129.

[plw037-B18] Kessler D Clark DG Colquhoun TA Baldwin IT. 2013 Defensive function of herbivore-induced plant volatile emissions in nature. Ecology Letters 16:299–306.23173705

[plw037-B19] Kudo G. 2003 Anther arrangement influences pollen deposition and removal in hermaphrodite flowers. Functional Ecology 17:349–355.

[plw037-B20] López-Portillo J Eguiarte LE Montaña C. 1993 Nectarless honey mesquites. Functional Ecology 7:452–461.

[plw037-B21] Makino TT Sakai S. 2007 Experience changes pollinator responses to floral display size: from size-based to reward-based foraging. Functional Ecology 21:854–863.

[plw037-B22] Makino TT Ohashi K Sakai S. 2007 How do floral display size and the density of surrounding flowers influence the likelihood of bumble bee revisitation to a plant? Functional Ecology 21:87–95.

[plw037-B23] McDonald JH. 2014 Nested anova In: McDonald JH Handbook of Biological Statistics, 3rd edn Baltimore, Maryland: Sparky House Publishing.

[plw037-B24] Mitchell RJ. 2004 Heritability of nectar traits: why do we know so little? Ecology 85:1527–1533.

[plw037-B25] Ohashi K Yahara T. 2002 Visit larger displays but probe proportionally fewer flowers: counterintuitive behaviour of nectar-collecting bumble bees achieves an ideal free distribution. Functional Ecology 16:429–503.

[plw037-B26] Ohashi K Thomson JD D'Souza D. 2007 Trapline foraging by bumble bees: IV. Optimization of route geometry in the absence of competition. Behavioral Ecology 18:1–11.

[plw037-B27] Ordano M Ornelas JF. 2005 The cost of nectar replenishment in two epiphytic bromeliads. Journal of Tropical Ecology 21:541–547.

[plw037-B28] Peter CI Johnson SD. 2008 Mimics and magnets: the importance of color and ecological facilitation in floral deception. Ecology 89:1583–1595. 18589523 10.1890/07-1098.1

[plw037-B29] R Core Team. 2014 R: a language and environment for statistical computing. Vienna, Austria: R Foundation for Statistical Computing.

[plw037-B30] Real LA. 1981 Uncertainty and pollinator-plant interactions: the foraging behavior of bees and wasps on artificial flowers. Ecology 62:20–26.

[plw037-B31] Real LA, ed. 1983 Pollination biology. New York, USA: Academic Press.

[plw037-B32] Renner S. 2006 Rewardless flowers in the angiosperms and the role of insect cognition in their evolution In: Waser NM Ollerton J eds. Plant-pollinator interactions: from specialization to generalization. USA: University of Chicago Press, 123–144.

[plw037-B33] Schiestl FP. 2010 The evolution of floral scent and insect chemical communication. Ecology Letters 13:643–656. 20337694 10.1111/j.1461-0248.2010.01451.x

[plw037-B34] Smithson A Gigord LD. 2003 The evolution of empty flowers revisited. American Naturalist 161:537–552. 10.1086/368347 12776883

[plw037-B35] Thomson JD Maddison WP Plowright RC. 1982 Behavior of bumblebee pollinators of *Aralia hispida* Vent. (Araliaceae). Oecologia 54:326–336. 10.1007/BF00380001 28309956

[plw037-B36] Thostesen A Olesen J. 1996 Pollen removal and deposition by specialist and generalist bumblebees in *Aconitum septentrionale.* Oikos 77:77–84.

[plw037-B37] Willmer P. 2011 Pollination and floral ecology. New Jersey, USA: Princeton University Press.

