# Supplementary material for: Foraging responses of bumble bees to rewardless floral patches: importance of within-plant variance in nectar presentation
Source: AoB Plants. 2016 Jul 11;8:plw037. doi: 10.1093/aobpla/plw037 (PMC4940503; doi:10.1093/aobpla/plw037)
Supplement: Supplementary Data [file supp_8_plw037_index.html]

Foraging responses of bumble bees to rewardless floral patches: importance of within-plant variance in nectar presentation — Supplementary Data 

# Foraging responses of bumble bees to rewardless floral patches: importance of within-plant variance in nectar presentation

## Supplementary Data

files

- Supplementary Data - docx file
- Supplementary Data - docx file
- Supplementary Data - docx file
- Supplementary Data - docx file
- Supplementary Data - docx file
