# Supplementary material for: Foraging responses of bumble bees to rewardless floral patches: importance of within-plant variance in nectar presentation
Source: AoB Plants. 2016 Jul 11;8:plw037. doi: 10.1093/aobpla/plw037 (PMC4940503; doi:10.1093/aobpla/plw037)
Supplement: Supplementary Data [file supp_plw037_aobplants-15300-s01.docx]

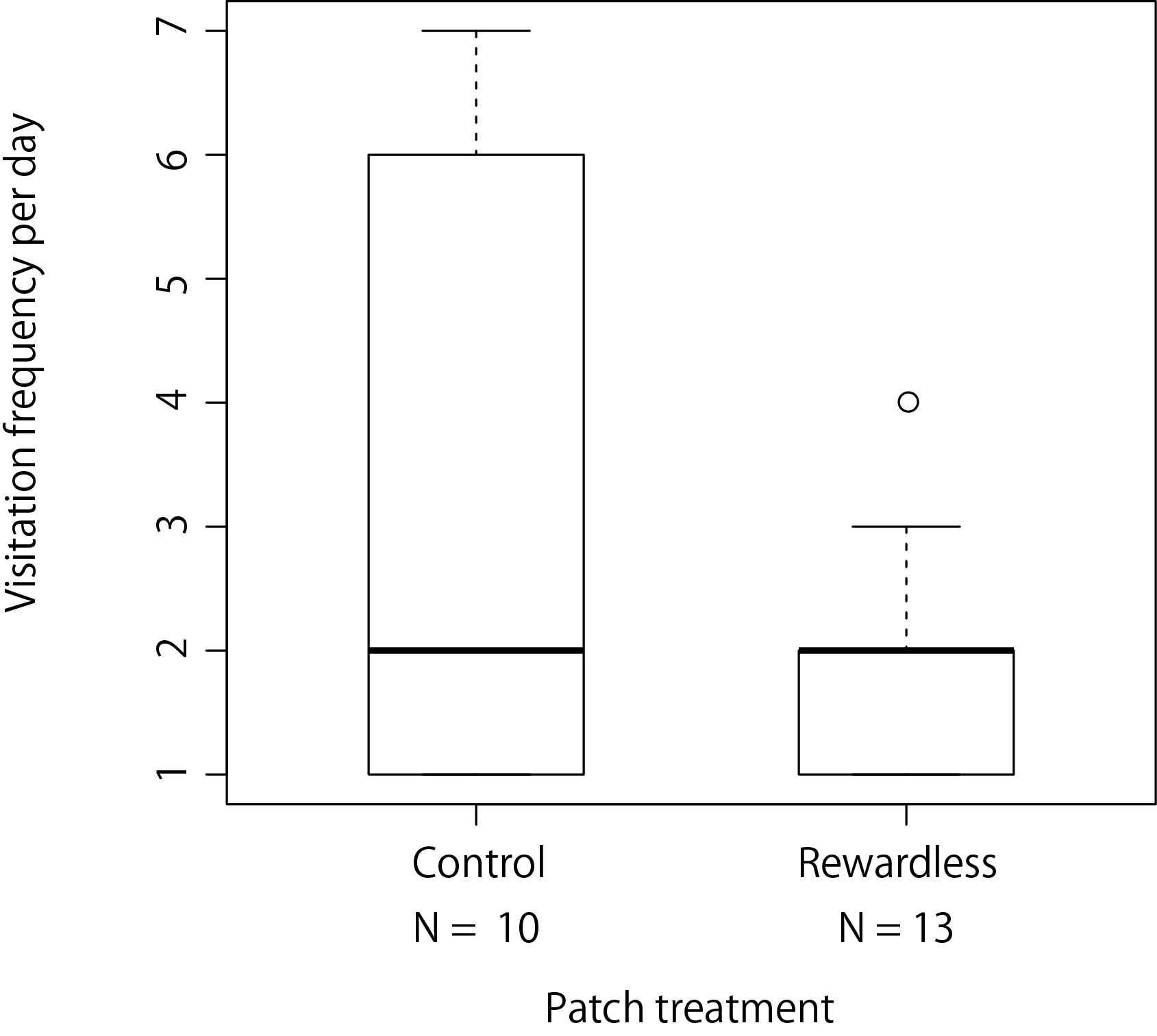


Figure S1. Boxplots of the visitation frequency of identified bumble bees to the control and rewardless patches. The lower ends of the boxes represent the first quartiles and the upper the third quartiles, the segments inside the boxes indicate medians, whiskers above the boxes indicate the maximums within the range of 1.5 times of interquartile range from the upper quartiles, and an outlier is represented by an open circle. According to a GLMM assuming a Poisson error distribution in which date and bee ID were treated as random factors and nectar treatment was included as a fixed factor, re-visitation frequencies were lower at the rewardless patches at marginal significance (*z* = −1.70, *P* = 0.089). Identified bumble bees that visited the control and rewardless patches in the grassland site were recorded for 2,190 min on 5 days (i.e., 438 ± 40 (mean ± SD) min per day) between 24 and 30 August 2011. Identification was made by marking of unique combination of colours using marker paint. A total of 51 bumble bees were identified between 9 and 28 August, and eight bumble bees were observed during the observation.
