# Supplementary material for: Foraging responses of bumble bees to rewardless floral patches: importance of within-plant variance in nectar presentation
Source: AoB Plants. 2016 Jul 11;8:plw037. doi: 10.1093/aobpla/plw037 (PMC4940503; doi:10.1093/aobpla/plw037)
Supplement: Supplementary Data [file supp_plw037_aobplants-15300-s02.docx]

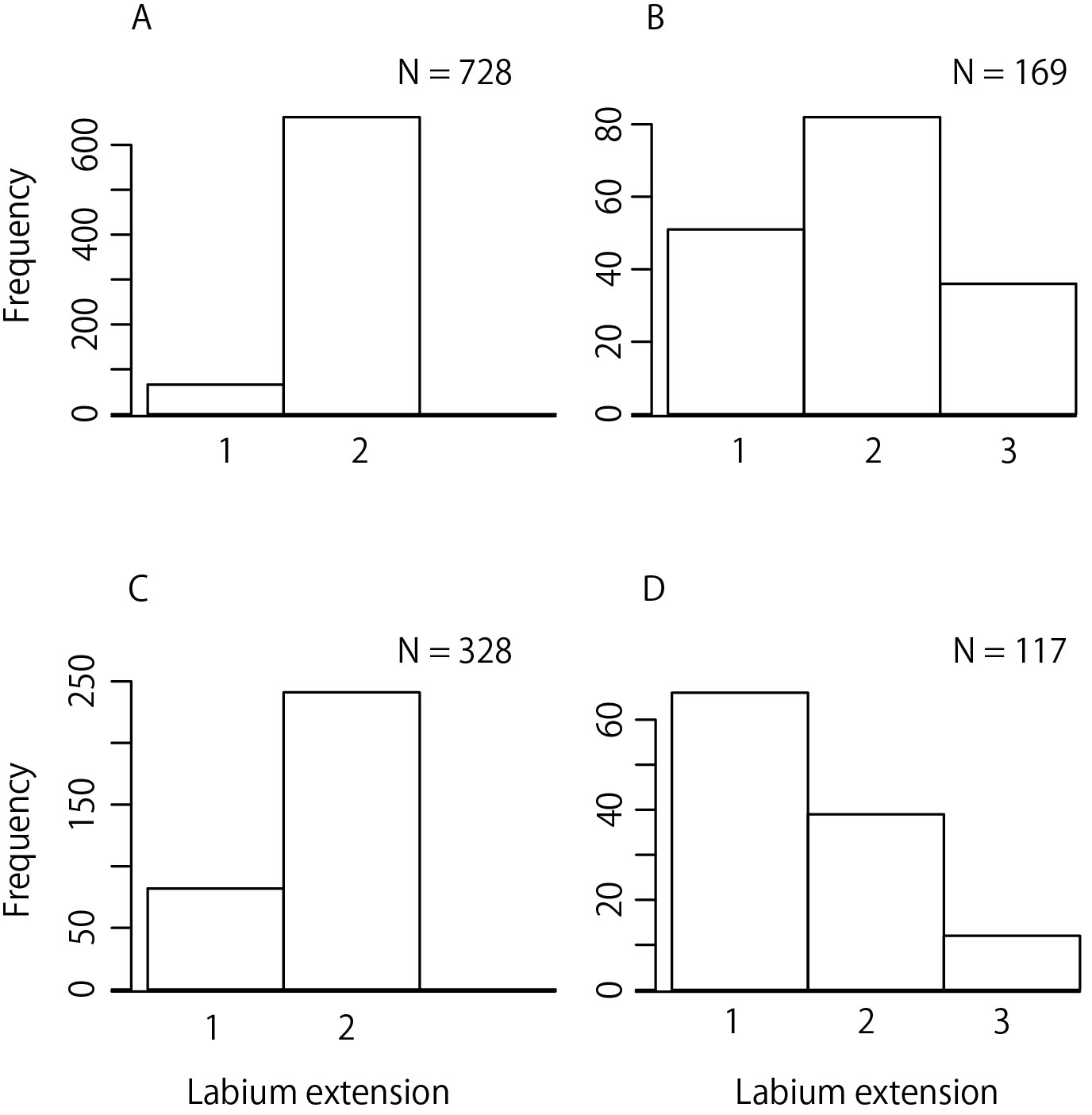


Figure S2. The number of labium extension as bumble bees probed nectaries through petals within flowers at control (A) and rewardless (B) patches in the forest site, and control (C) and rewardless (D) patches in the grassland site.

We could follow the probing behaviour by observing back and forth movement of bumble bees during foraging in each flower. Most of the bumble bees extended their labium once for each of the two nectaries (i.e., in this case, the number of labium extension would be two). According to the GLMM assuming a Poisson error distribution, in which site and nectar treatment were included as fixed factors and inflorescence ID was treated as a random factor, the number of labium extension was larger in the forest site (*z* = −2.77, *P* = 0.006). The effect of nectar treatment was excluded by AIC.
