# Supplementary material for: Foraging responses of bumble bees to rewardless floral patches: importance of within-plant variance in nectar presentation
Source: AoB Plants. 2016 Jul 11;8:plw037. doi: 10.1093/aobpla/plw037 (PMC4940503; doi:10.1093/aobpla/plw037)
Supplement: Supplementary Data [file supp_plw037_aobplants-15300-s03.docx]

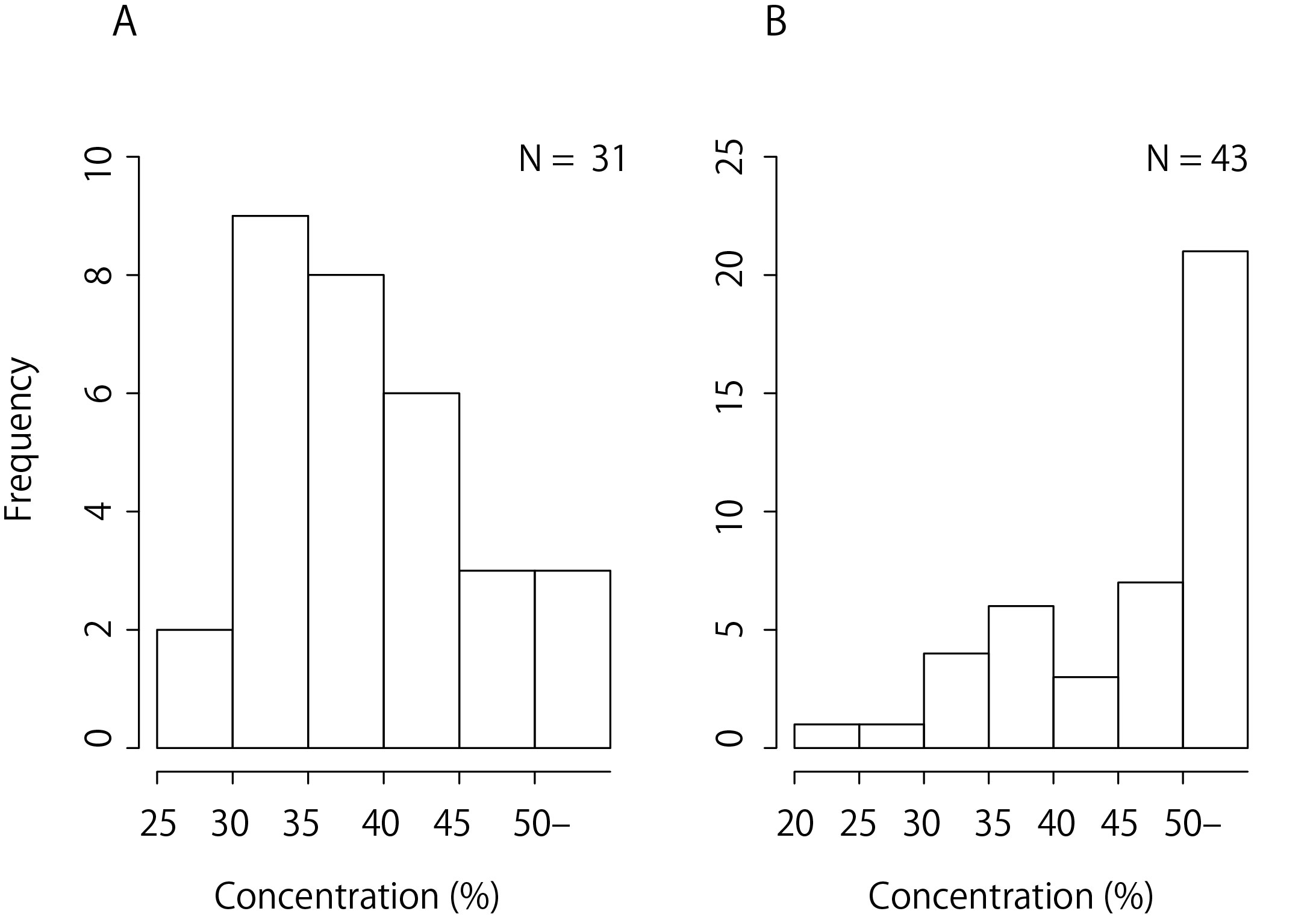


Figure S3. Variation in the nectar sugar concentration of *Aconitum sachalinense* flowers in the forest (A) and grassland (B) sites. Nectar sugar concentration was higher in the grassland site (Wilcoxon rank sum test, *W* = 332.5, *P* <0.01). In total, nectar of 31 and 43 flowers were measured in the forest and grassland sites, respectively, on 7 days during 14 August to 7 September 2010 using a refractometer (Eclipse Professional Grade Optical Hand Held Refractometer, Bellingham + Stanley Ltd., U.K.).
