# Supplementary material for: Foraging responses of bumble bees to rewardless floral patches: importance of within-plant variance in nectar presentation
Source: AoB Plants. 2016 Jul 11;8:plw037. doi: 10.1093/aobpla/plw037 (PMC4940503; doi:10.1093/aobpla/plw037)
Supplement: Supplementary Data [file supp_plw037_aobplants-15300-s04.docx]

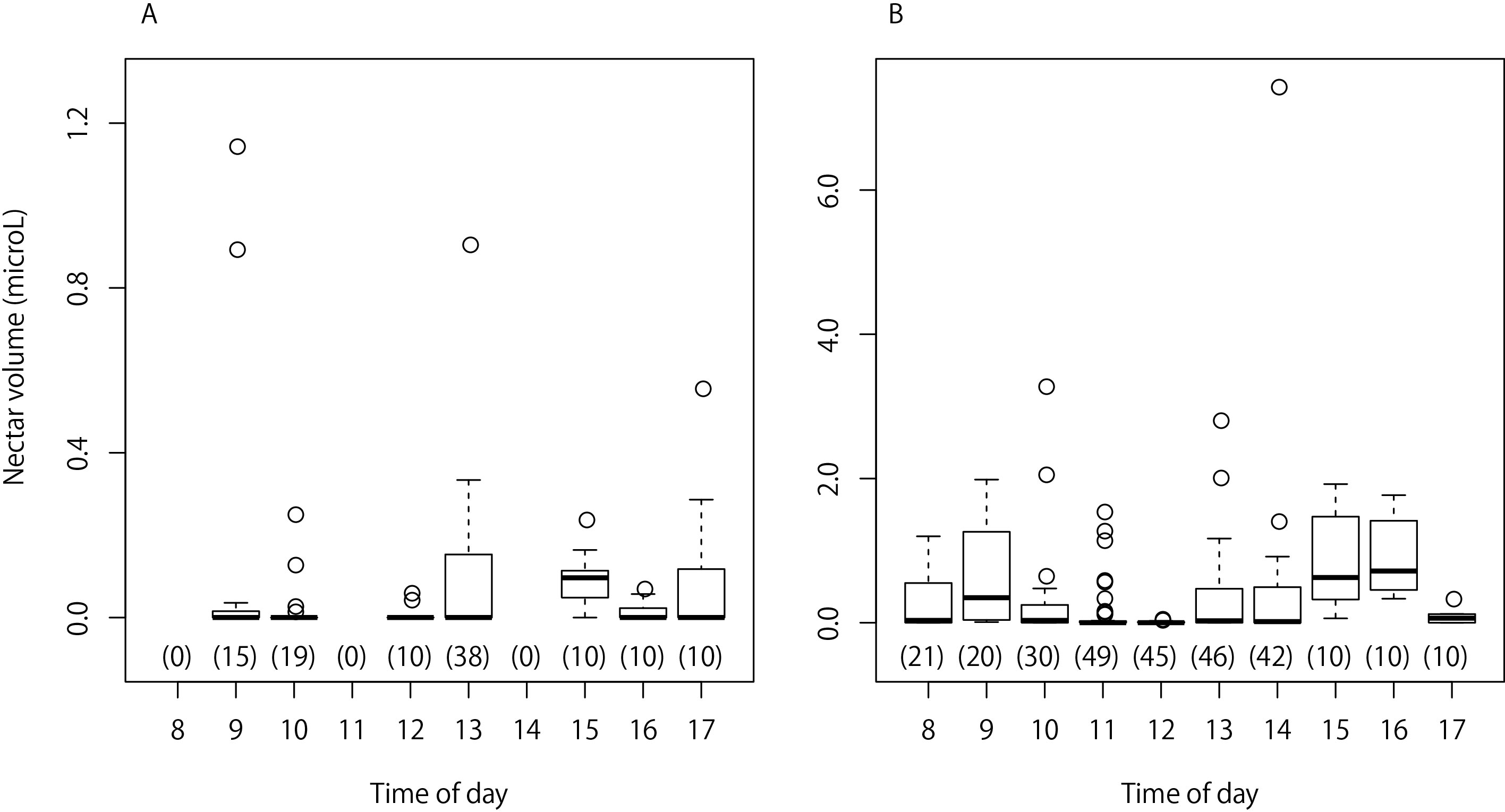


Figure S4. Diel variation in nectar standing crop of intact (i.e. unmanipulated) flowers of *Aconitum sachalinense* in the forest (A) and grassland (B) sites. Figures in parentheses represent the number of samples. Measurements were made on 7 or 11 days during the flowering season of 2010, 2011, and 2015 in the forest and grassland sites, respectively. Flowers were randomly selected, without controlling floral sexual phase.

The average nectar standing crop was 0.07 ± 0.18 (mean ± SD) µL (*N* = 112) and 0.28 ± 0.66µL (*N* = 283) in the forest and grassland sites, respectively. Among these flowers, about 60% (67 flowers) and 42% (118 flowers) produced no collectable nectar in the forest and grassland sites, respectively.
