# Supplementary material for: Foraging responses of bumble bees to rewardless floral patches: importance of within-plant variance in nectar presentation
Source: AoB Plants. 2016 Jul 11;8:plw037. doi: 10.1093/aobpla/plw037 (PMC4940503; doi:10.1093/aobpla/plw037)
Supplement: Supplementary Data [file supp_plw037_aobplants-15300-s05.docx]

Table S1. Raw data of visitation frequency of identified bumble bees.

| Date | Bee ID | Marking date | Control | Rewardless |
| --- | --- | --- | --- | --- |
| 2011/8/24 | BBG | 2011/8/21 | 6 | 4 |
| 2011/8/24 | BBPi | 2011/8/21 | 0 | 1 |
| 2011/8/24 | RRG | 2011/8/23 | 2 | 2 |
| 2011/8/26 | RRG | 2011/8/23 | 0 | 1 |
| 2011/8/29 | GGY | 2011/8/28 | 1 | 0 |
| 2011/8/29 | RRG | 2011/8/23 | 7 | 1 |
| 2011/8/29 | YGY | 2011/8/28 | 0 | 2 |
| 2011/8/29 | YYG | 2011/8/28 | 2 | 2 |
| 2011/8/29 | YYPu | 2011/8/28 | 1 | 1 |
| 2011/8/30 | GGY | 2011/8/28 | 0 | 1 |
| 2011/8/30 | RRG | 2011/8/23 | 6 | 3 |
| 2011/8/30 | YGY | 2011/8/28 | 2 | 2 |
| 2011/8/30 | YPiY | 2011/8/25 | 1 | 2 |
| 2011/8/30 | YYG | 2011/8/28 | 1 | 1 |

Identified bumble bees that visited the control and rewardless patches in the grassland site were recorded during 2190 min on 5 days (i.e., 438 ± 40 (mean ± SD) min per day on average) between 24 and 30 of August 2011. Identification was made by markings of unique combination of colours using marker paint. A total of 51 bumble bees were marked between 9 and 28 of August, and eight individuals were observed during the observation.
